# Direct Formation of Sub-Micron and Nanoparticles of a Bioinspired Coordination Polymer Based on Copper with Adenine

**DOI:** 10.3390/polym9110565

**Published:** 2017-11-01

**Authors:** Verónica G. Vegas, Marta Villar-Alonso, Carlos J. Gómez-García, Félix Zamora, Pilar Amo-Ochoa

**Affiliations:** 1 Departamento de Química Inorgánica, Universidad Autónoma de Madrid, 28049 Madrid, Spain; veronica.garciav@uam.es (V.G.V.); marta.villara@estudiante.uam.es (M.V.-A.); 2 Departamento de Química Inorgánica, Instituto de Ciencia Molecular (ICMol), Parque Científico, Universidad de Valencia, Catedrático José Beltrán, 2, Paterna, 46980 Valencia, Spain; carlos.gomez@uv.es; 3 Instituto de Física de la Materia Condensada (IFIMAC), Universidad Autónoma de Madrid, 28049 Madrid, Spain; 4 Institute for Advanced Research in Chemical Sciences (IAdChem), Universidad Autónoma de Madrid, 28049 Madrid, Spain

**Keywords:** coordination polymers, surfactants, nanoparticles

## Abstract

We report on the use of different reaction conditions, e.g., temperature, time, and/or concentration of reactants, to gain control over the particle formation of a bioinspired coordination polymer based on copper(II) and adenine, allowing homogeneous particle production from micro- to submicro-, and up to nano-size. Additionally, studies on this reaction carried out in the presence of different surfactants gives rise to the control of the particle size due to the modulation of the electrostatic interactions. Stability of the water suspensions obtained within the time and pH has been evaluated. We have also studied that there is no significant effect of the size reduction in the magnetic properties of the Cu(II)-adenine coordination polymer.

## 1. Introduction

Coordination Polymers (CPs) encompass a large group of compounds, formed by central metal ions and ligands linked by coordination bonds. Depending on the selected building blocks, CPs give rise to mono-, bi-, or three-dimensional structures. Additionally, one- or two-dimensional CPs can organize their three-dimensional networks using supramolecular interactions (such as hydrogen bonds) or van der Waals forces. The constituent building blocks of the CPs are also determinants in their physical and chemical properties [1,2,3]. Indeed, CPs are a fundamental source of multifunctional materials that may combine several physical properties, such as emission and magnetism, or emission and electrical conductivity [1]. Recently, a new family of CPs fusing bioinspired molecules, such as amino acids or peptides, and nucleobases as ligands has emerged. Although these bioinspired CPs could present new potential applications, the number of examples is still limited [4,5,6,7,8,9,10,11,12]. Indeed, nucleobases have intrinsic self-assembly abilities that can be used to direct the structure and function of CPs. This molecular self-assembly capability requires the establishment of intermolecular hydrogen bonds between complementary nucleobases or between nucleobases and others organic/inorganic ligands. Therefore, these new bioinspired CPs may show promising biological and/or biomedical interest [13]. Moreover, the production of these materials at the nanoscale will enhance their potential applications in biomedicine, e.g., traversing the cellular membrane [13].

Another important aspect in the nanomaterials area is that the physical properties may vary with respect to the bulk material as a consequence of this size reduction; therefore, methods for controlling the size distribution of nanomaterials are key prerequisites to gain access to a plethora of interesting physical properties and size-dependent phenomena inherent to nanomaterials. 

In this regard, coordination polymers have, among other interesting advantages, the facility to be nanoprocessed [14,15,16,17,18]. In recent years, a large number of nanostructured coordination polymers have been obtained through different processes, such as interfacial reactions, hydro-solvothermal reactions, microemulsions, or using ultrasound [19] and the use of surfactants [20]. However, probably the simplest procedure based on fast precipitation controlling the concentration of the reactants or the addition of a poor solvent to the reaction medium, can also be enough to produce a reduction from the microscale to the nanoscale size, which it is little developed.

Finally, it is worth mentioning that, despite the number of studies focusing on the production of nanostructures of CPs with ligands of biological interest still being very limited, in most of the cases, the properties of these compounds have been studied at the bulk scale or even without an analysis of material size.

Therefore, the synthesis, size, morphology, and stability control of nanostructures based on coordination polymers bearing bio-inspired ligands is a subject of research interest because it will allow the preparation of new biocompatible (nano)particles able to penetrate at the cellular level, acting as liberators or transporters of substances of biological or pharmacological interest [21] and extend their possible uses to create multifunctional biologically-compatible nanodevices [22], catalytic nanoparticles [23], antimicrobial drugs [24], nanocarriers [13], or anticancer chemotherapy drugs [25].

Herein, we describe easy ways to control the size, from the micro- to the sub-micron-scale, of a bioinspired coordination polymer based on copper(II) with adenine (Ad) [26]. We have studied the reaction conditions (such as reaction time, concentration, and surfactants) in order to analyze their effects on the resulting size of the material. Additionally, we have studied the effect of the presence of ionic, non-ionic, and cationic surfactants in this reaction to modify the material size by selective interactions [27]. Moreover, stability studies over the time and in a pH range have also been carried out in order to determine/evaluate the shape and size of the changes [27]. Finally, the study of the magnetic properties comparing micro- and submicron particles has also been conducted.

## 2. Experimental Section

### 2.1. Materials and Methods

All reagents and solvents were purchased from standard chemical suppliers and used as received.

Infrared (IR) spectra were recorded on a PerkinElmer 100 spectrophotometer (PerkinElmer Inc., Waltham, MA, USA) using a PIKE Technologies MIRacle Single Reflection Horizontal ATR Accessory from 4000–600 cm^−1^. Elemental analysis was performed on an LECO CHNS-932 Elemental Analyzer (LECO Corporation, St. Joseph, MI, USA). Powder X-ray diffraction has been collected using a Diffractometer PANalyticalX'Pert PROθ/2θ (PANanalytical, Almelo, The Netherlands) primary monochromator and detector with fast X’Celerator. Theoretical X-ray powder diffraction patterns were calculated using Mercury Cambridge Structural Database (CSD) version 3.6 software from the Crystallographic Cambridge Data Base (CCDC). The samples have been analysed with scanning θ/2θ. Magnetic measurements were done in a Quantum Design MPMS-XL-5 SQUID magnetometer (San Diego, CA, USA) in the 2–300 K temperature range with an applied magnetic field of 0.1 T in a water suspension of the sample. The susceptibility data were corrected for the sample holders previously measured under the same conditions, and for the diamagnetic contributions as deduced by using Pascal´s constant tables [28].

FESEM images were recorded on a Philips XL30 S-FEG (Eindhoven, The Netherlands) field emission scanning electron microscope.

SEM sample preparation of {[Cu_2_(μ_3_-adeninato)_2_(μ-Hadip)_2_]}_n_ (**1n**): 0.017 g of **1n** were suspended in 21 mL of water. 20 μL of this suspension was diluted with 100 μL of Milli-Q water. Then, 10 μL of the diluted suspension was deposited on glass substrates by drop-casting and allowed to adsorb for 15 min at room temperature. The remaining suspension was removed blowing with an argon flow. 

SEM samples preparation of surfactant-mediated {[Cu_2_(μ_3_-adeninato)_2_(μ-Hadip)_2_]}_n_ (**1n**): 0.017 g of **1n** were suspended in 21 mL of water. 20 μL of this suspension was diluted with 100 μL of Milli-Q water. Then, 10 μL of the diluted suspensions were deposited on glass substrates by drop-casting and allowed to adsorb for 15 min at room temperature. The remaining suspensions were removed by blowing with an argon flow.

Atomic force microscope (AFM) images were acquired in dynamic mode using a Nanotec Electronica system (NANOTEC, Madrid, Spain) operating at room temperature in ambient air conditions. For AFM measurements, Olympus cantilevers were used with a nominal force constant of 0.75 N/m and a resonance frequency of about 70 kHz. The images were processed using WSxM [29]. The surfaces used for AFM were SiO_2_, 300 nm in thickness (IMS Company). SiO_2_ surfaces were sonicated in an ultrasound bath at 37 KHz and 380 Watts, for 15 min in acetone, 15 min in 2-propanol, and then dried under an argon flow.

AFM sample preparation of {[Cu_2_(μ_3_-adeninato)_2_(μ-Hadip)_2_]}_n_ (**1n**): 0.017 g of **1n** were suspended in 21 mL of water. 100 μL of this suspension was diluted with 1 mL of Milli-Q water. Then, 15 μL of the diluted suspension was deposited on SiO_2_ substrates by drop-casting and allowed to adsorb for 15 min at room temperature. The remaining suspension was removed by blowing with an argon flow.

Size distribution and surface charge of the submicroparticles were measured by Dynamic Light Scattering (DLS), using a ZetasizerNano-ZS instrument (Malvern Instruments, Malvern, UK), and a Vasco 1 particle size analyser of Cordouan Technologies. The size range limit (diameter) is 0.3 nm to 10 μm (3.8 nm to 10 μm for zeta-potential). Note: the diameter measured by DLS is the hydrodynamic diameter. The samples were comprised of aqueous dispersions of the submicroparticles in distilled water. All samples were diluted to obtain an adequate submicroparticle concentration. To study the influence of pH on zeta-potential, the pH of sample was modified by HCl (0.01 M) solution (pH = 5.0–5.9).

### 2.2. Synthesis

#### 2.2.1. Synthesis of {[Cu_2_(μ_3_-adeninato)_2_(μ-Hadip)_2_]}_n_ (**1m**)

Green microcrystals of compounds **1m** were prepared by slow diffusion of a methanolic solution (5 mL) of 0.2 mmol of adipic acid (0.0295 g) into an aqueous solution of 0.2 mmol of Cu(NO_3_)_2_·3H_2_O (0.0483 g, 5 mL) and 0.2 mmol of adenine (0.0273 g, 20 mL) as previously reported [26] (0.096 g, 70% yield based on metal). Anal. Calcd. (%). (found) for C_22_H_26_Cu_2_N_10_O_8_ (685.62 g/mol): C, 38.54 (38.33); H, 3.82 (4.02); N, 20.43 (20.06); IR (cm^−1^, KBr pellet): 3410 (s), 3350 (m), 3210 (m), 2940 (m), 1710 (m), 1655 (s), 1615 (m), 1580 (s), 1538 (m), 1466 (w), 1452 (w), 1400 (m), 1385 (w), 1344 (w), 1316 (m), 1280 (w), 1238 (w), 1205 (m), 1155 (m), 1037 (w), 1014 (w), 991 (w), 921 (w), 899 (w), 866 (w), 792 (m), 740 (w), 649 (m), 600 (m). X-ray power diffraction confirmed its purity and structure.

#### 2.2.2. Synthesis of Submicron {[Cu_2_(μ_3_-adeninato)_2_(μ-Hadip)_2_]}_n_ (**1n**)

A mixture of an aqueous solution (25 °C, 4 mL) of adipic acid (H_2_adip) (0.2 mmol, 0.0295 g), an aqueous solution (25 °C, 4 mL) of Cu(NO_3_)_2_·3H_2_O (0.2 mmol, 0.0483 g) and an aqueous solution (25 °C, 13 mL) of adenine (0.2 mmol, 0.0273 g) was stirred for 5 min at 40 °C (pH = 5.8). After that, the resulting deep-blue suspension was allowed to stand at 25 °C for 20 h and during this time is turned onto a green suspension. Finally, the suspension was centrifuged for 5 min at 10000 rpm. The green solid obtained was washed four times with 1 mL of water and dried under vacuum (0.017 g, 13% yield). Anal. Calcd. (%) (found) for C_22_H_26_Cu_2_N_10_O_8_ (685.62 g/mol): C, 38.54 (38.90); H, 3.82 (3.91); N, 20.43 (19.65). IR selected data $\bar{\nu }$ (cm^−1^): 3345 (s), 3172 (s), 2962 (m), 1718 (m), 1666 (s), 1619 (s), 1577 (s), 1538 (m), 1463 (m), 1452 (m), 1398 (m), 1342 (m), 1315 (m), 1280 (m), 1213 (m), 1155 (m), 1079 (w), 937 (w), 900 (w), 792 (m), 775 (m), 649 (m). X-ray powder diffraction confirmed the structure of the sample.

#### 2.2.3. Surfactant-Mediated Syntheses of Submicron {[Cu_2_(μ_3_-adeninato)_2_(μ-Hadip)_2_]}_n_ (**1n**)

Stock solutions of 1, 5, and 15 mM sodium dodecyl sulfate (SDS), cetyltrimethylammonium bromide (CTAB), and 1, 5, and 15 g/L of polyethylene glycol (P_123_; M = 5800) were prepared in water. 

For each concentration of surfactant the synthetic procedure is as follows:

Cu(NO_3_)_2_·3H_2_O (0.2 mmol, 0.0483 g) and adenine (0.2 mmol, 0.0273 g) were dissolved in 4 and 13 mL of the stock surfactant solution, respectively, and both solutions mixed. Then, adipic acid (H_2_adip) (0.2 mmol, 0.0295 g) was dissolved in 4 mL of the stock surfactant solution at 25 °C and added to the initial Cu(NO_3_)_2_·3H_2_O and adenine solution. After that, the mixture was stirred for 5 min at 40 °C, and the resulting deep blue suspensions were then stored at 25 °C for 20 h. During this time it turned deep purple for CTAB, purple for SDS, and green for P_123_. Finally, each suspension was centrifuged for 10 min at 5000 rpm and the solids washed four times with 1 mL of water each time and dried under vacuum.

## 3. Results and Discussion

**Figure 1:**
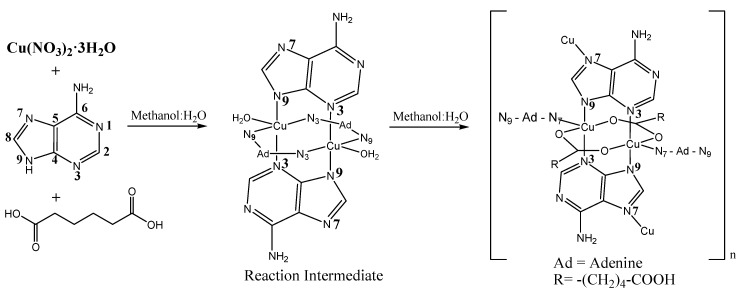
Synthesis scheme of {[Cu_2_(μ_3_-adeninato)_2_(μ-Hadip)_2_]}_n_ (**1m**) through the intermediate [Cu_2_(μ-N3,N9-adeninato)_4_(H_2_O)_2_].

Formation of coordination polymers typically consists of a one-step process based on precipitation when a poor solvent is used or when the concentration of the CP is suitable. This simple procedure can directly originate the nanostructuration of the material. The synthesis and structure of compound {[Cu_2_(μ_3_-adeninato)_2_(μ-Hadip)_2_]}_n_ 5H_2_O (**1m**) was previously reported [26]. However, in this case the preparation of **1m** seems to take place in a two-step process in which, first, the dinuclear complex [Cu_2_(μ-N3,N9-adeninato)_4_(H_2_O)_2_] seems to be formed in suspension (Figure 1), as is shown by its IR spectra (Table S1) and evolves to **1m** [30].

**Figure 2:**
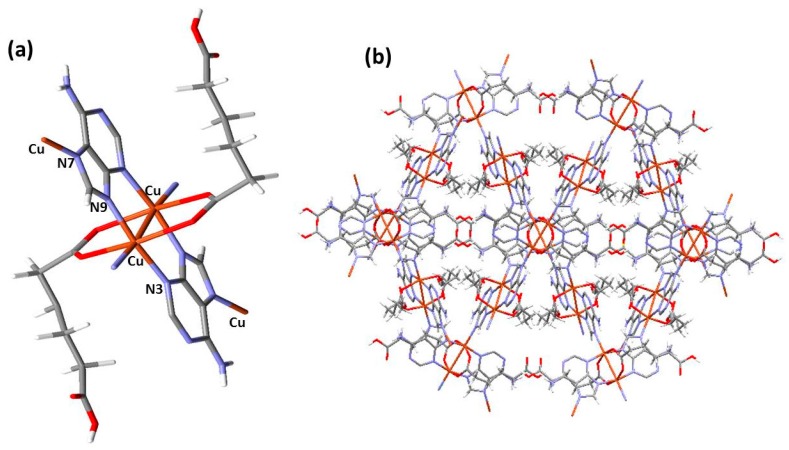
Different views of the structure of {[Cu_2_(μ_3_-adeninato)_2_(μ-Hadip)_2_]}_n_ (**1m**) (ref. code KEBPOY from the Cambridge Structural Database, CSD) [26]. (**a**) Fragment of the repetition unit of compound **1m** that shows the paddle-wheel motif. To clarify nitrogen atoms are in blue, copper atoms are in orange, carbon atoms are in grey, oxygen atoms are in red, and hydrogens atoms are in white. (**b**) The perspective view of the overall 3D structure. The structural disorder has been omitted in the drawing for clarity.

The interesting structure of {[Cu_2_(μ_3_-adeninato)_2_(μ-Hadip)_2_]}_n_ consists of a three-dimensional coordination polymer where the Cu(II) centers are linked by tridentate adenine ligands (N3, N7, N9) and the carboxylate anions act as bidentate ligands (μ-kO1:kO2). Interestingly, the structure shows the presence of free amino groups corresponding to the un-coordinated adenine N(6)H_2_ and free carboxyl groups placed within the channels (Figure 2) [26]. However, despite its interesting structural features and potential applications, its potential nano-structuration has not yet been studied.

### 3.1. Effect of Temperature, Reaction Time and Concentration in the Size and Shape of {[Cu_2_(μ_3_-adeninato)_2_(μ-Hadip)_2_]}_n_ (**1n**)

**Figure 3:**
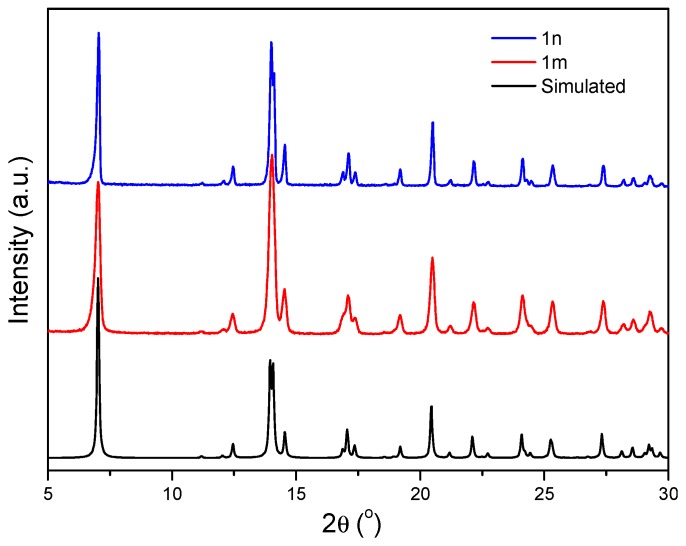
X-ray powder diffraction patterns of simulated from DRX data from the single crystal structural elucidation of {[Cu_2_(μ_3_-adeninato)_2_(μ-Hadip)_2_]}_n_ (black line), (**1m**) microcrystalline compound (red line), and (**1n**) submicroparticles (blue line).

An analysis of the green single crystals of **1m** obtained following the previously-reported synthesis carried out by slow diffusion of a methanolic solution of adipic acid (H_2_adip) into an aqueous Cu(NO_3_)_2_·3H_2_O and adenine solution in a 1:1:1 stoichiometry (Figure 1) [26] give rise to the isolation of octahedral micron size crystals formed by slow liquid-liquid diffusion after several days (Figure 3).

We have first decided to analyse the effect of the temperature and reaction time over the particle size [30]. In our approach, adenine, adipic acid, and copper(II) are dissolved in water by heating during some minutes and, later, the solution is stored for 20–24 h at 20 °C to produce {[Cu_2_(μ_3_-adeninato)_2_(μ-Hadip)_2_]}_n_. During the heating the initial blue solution turned into a green suspension indicating a change in the coordination sphere of the Cu(II) cation.

**Figure 4:**
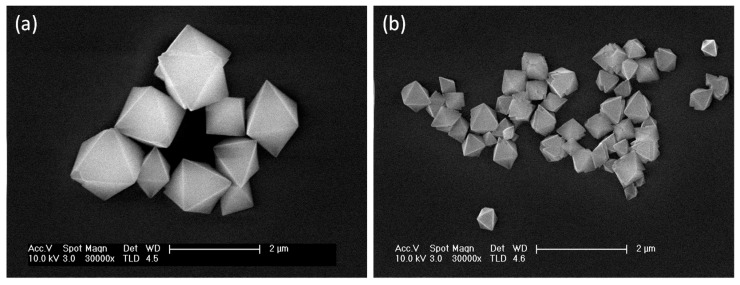
FESEM images of {[Cu_2_(μ_3_-adeninato)_2_(μ-Hadip)_2_]}_n_: (**a**) octahedral 1.2 ± 0.3 micrometre particles obtained from experiment *a*; and (**b**) octahedral 403 ± 130 nm particles obtained from experiment *c*.

The two syntheses carried out upon heating at 40 and 90 °C for 15 min afforded octahedral particles, where the size of those obtained at 90 °C had dimensions of ca. 1.2 ± 0.3 μm (Table 1, experiment *a*, Figure 4a), while a significant shortening in size of 381 ± 63 nm (Table 1, experiment *b*, Figure 4b) is observed when heating at 40 °C. Other experiments conducted at 40 °C and varying the reaction time do not show significant changes in the octahedral sizes (Table 1, experiments *b* and *c*). Similarly, experiments carried out at higher concentrations do not show significant variations in both shape and size (Table 1, experiments *d*–*h*).

Therefore, it seems that the reaction temperature is critical to produce a significant enhancement in the size of the particles. In view of the low yield obtained in experiment *a* (7% yield), we postulated that an increase in the temperature of the process (90 °C), could cause partial rupture of the intermediate dimer or partial esterification of the adipic acid [31] (Figure 1), reducing the concentration of the precursors, which would favour an increase in particle size.

**Figure 5:**
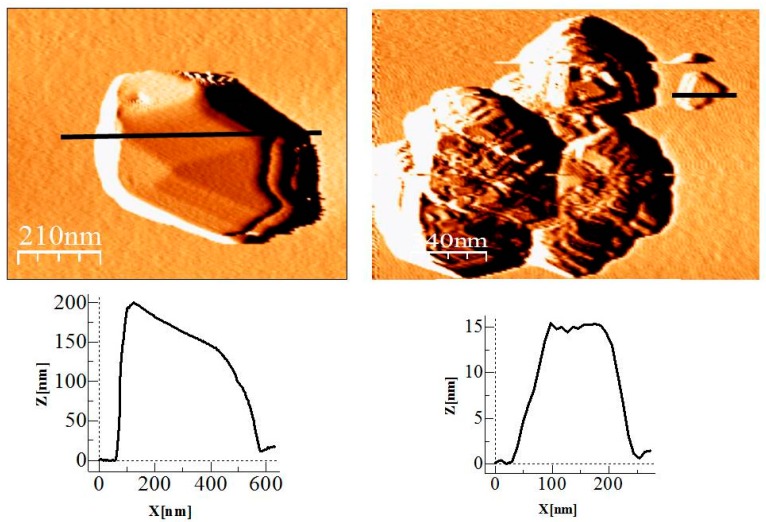
AFM images of {[Cu_2_(μ_3_-adeninato)_2_(μ-Hadip)_2_]}_n_ (**1n**) obtained in experiment *c*: Octahedral sub-micrometer and nanometer particles sizes (**top**); and the corresponding height profiles along the lines (**bottom**).

Atomic force microscopy (AFM) study has been used to complete the morphological characterization of the submicron octahedral **1n** particles, obtained in the experiment *c* (Figure 5).

**Figure 6:**
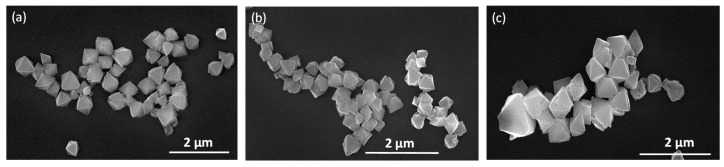
(**a**) FESEM images of sub-microparticles of compound **1n** with an average size of 403 ± 130 nm obtained heating at 40 °C for 5 min and leaving the solution 20 h at 25 °C; (**b**) **1n** sample stored for one week at 25 °C, with an average size of 467 ± 52 nm; and (**c**) **1n** sample stored for one month at 25 °C with an average size of 484 ± 28 nm. The means and standard deviations were calculated from values corresponding to 60 particles from FESEM images.

**Figure 7:**
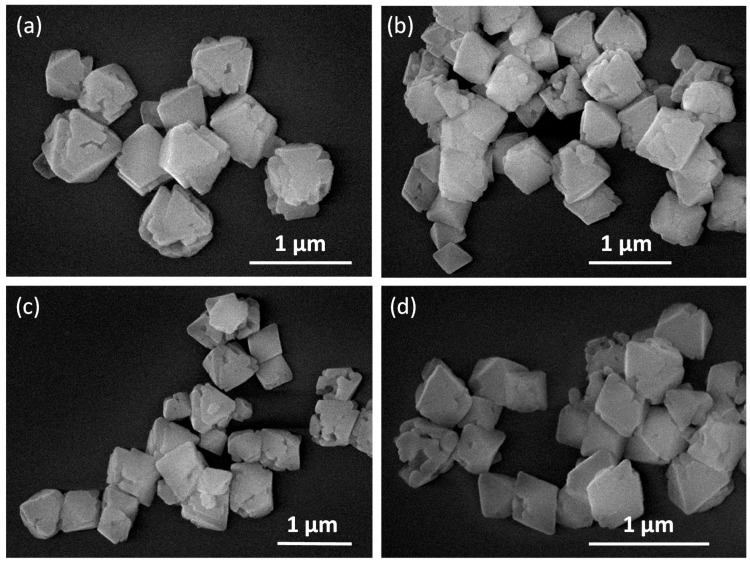
FESEM images showing the stability of **1n** obtained heating at 40 °C, 5 min and leaving the solution 20 h at 25 °C (*c* experimental conditions) versus pH: (**a**) pH = 5.5, average size of 534 ± 85 nm (**b**) pH = 5.7, average size of 480 ± 93 nm; (**c**) pH = 5.9, average size of 474 ± 85 nm; and (**d**) pH = 7, average size of 471 ± 84 nm. The means and standard deviations were calculated from values corresponding to 60 particles from FESEM images.

Additionally, stability studies of **1n** in aqueous solutions with the time and at physiological pHs have been carried out. This is an important aspect toward the potential use of these submicron-particles for instance under biological conditions. Thus, sub-micron-particles of **1n** at pH 5.8 are stable, retaining their morphological features, with sizes of ca. 400 nm, and shape, for at least one month (Figure 6). Moreover, **1n** is also stable at pHs between 5.5 and 7 without significant changes in size and shape modifications for 534–471 ± 84 (Figure 7). However, almost immediately, decomposition of **1n** is observed at pHs lower than 5.5 and higher than 7 (Figure S1).

### 3.2. Effect of Surfactant in the Size and Shape of {[Cu_2_(μ_3_-Adeninato)_2_(μ-Hadip)_2_]}_n_ (**1n**)

The surfactants usually have a template role in the particle formation and their action mechanism, in the final size and shape of the CP material, and may also depend on the surfactant character—ionic, cationic, or neutral—which can cause competition between electrostatic interactions such as surfactant and metal ion, hydrophobic interactions, and coordination interactions [32]. Most of them are affected by the surfactant concentration. Moreover, it has been previously described that the binding strength of the surfactant ligands to different facets of the nanocrystals can control the relative growth rates and then the geometry of the nanoparticles. Typically, the surfactants selectively absorbed on some facets of the nanocrystal nuclei and altered the surface free energy of the absorbed plane, leaving a faster growth speed of the unabsorbed plane and, thus, induced oriented growth to the final product [27].

**Figure 8:**
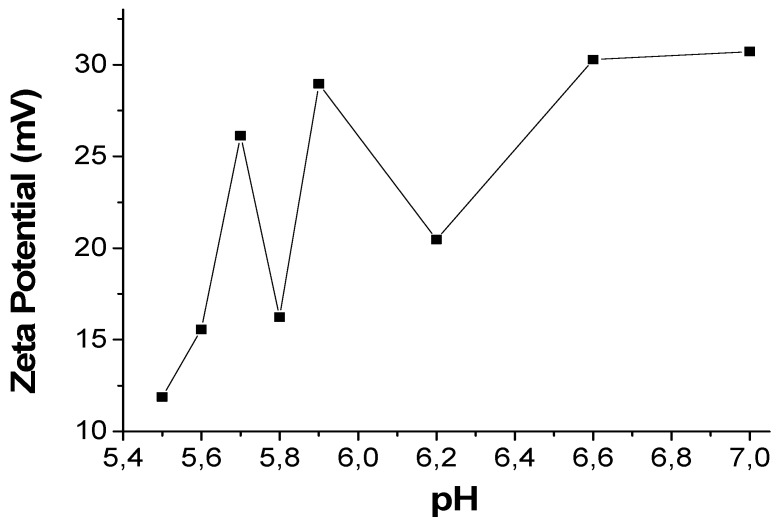
*Z*-potential values of **1n** at different pHs.

In order to study the size variation of compound **1n** in the presence of different types of surfactants at different concentrations, we have selected the surfactants looking for different types of interactions with {[Cu_2_(μ_3_-adeninato)_2_(μ-Hadip)_2_]}_n_. As we have already stated, the structure of {[Cu_2_(μ_3_-adeninato)_2_(μ-Hadip)_2_]}_n_ shows free acid groups corresponding to adipic acid and adenine ligands with free NH_2_ groups (Figure 1). In order to determine the net surface charge of {[Cu_2_(μ_3_-adeninato)_2_(μ-Hadip)_2_]}_n_, we have measured the *Z* potential of a **1n** suspension (Table 1, experiment *c*) at different pH values, from 5.5 to 7, in which **1n** is stable. The positive *Z* values (12–30 mV, Figure 8) obtained confirm that the net surface charge of **1n** presents a positive charge probably due to the protonation of amino groups [33].

Taking into account these considerations, the interaction of **1n** with different ionic surfactants, at suitable concentrations, could produce the following changes in its size: (i) an anionic surfactant, should change the *Z*-potential value from positive to negative, due to the electrostatic interactions and charge neutralization [34]; (ii) the electrostatic attractions between particle and surfactant should avoid the interaction between other particles avoiding the particle aggregation (hampering the particle growth); (iii) the interaction with a positively-charged surfactant should produce positive changes in the *Z*-potential and, therefore, electrostatic repulsions between the surfactant and the particle, which would favour the interactions leading aggregation between particles of **1n**, increasing the particle size; and (iv) the use of a neutral surfactant is not expected to produce any remarkable changes in *Z*-potential and, therefore, no significant changes in the particle size.

In order to corroborate these hypotheses, we have studied the interaction of **1n**, (obtained as in experiment *c*) with different surfactants; SDS (anionic), CTAB (cationic), and P_123_ (neutral) and at different concentrations (Table 2 and Table S2 and Figures S3–S7).

**Figure 9:**
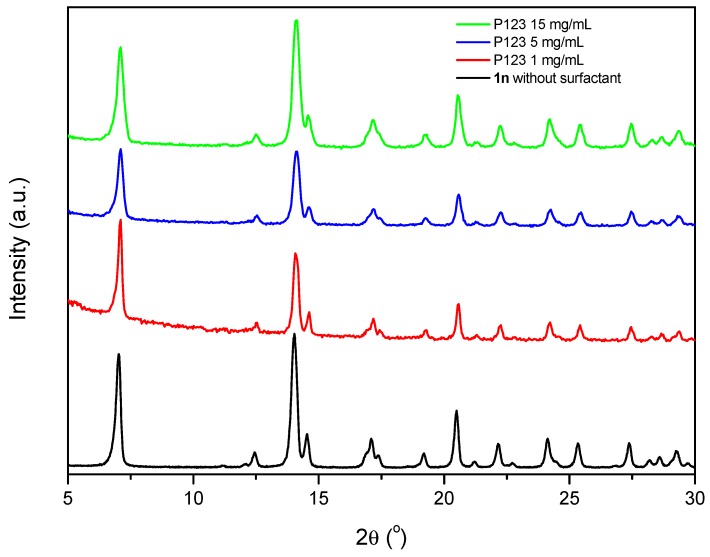
X-ray powder diffraction patterns of {[Cu_2_(μ_3_-adeninato)_2_(μ-Hadip)_2_]}_n_ (**1n**) (black) and the obtained products under different concentrations of the P_123_ surfactant.

First, the synthesis of {[Cu_2_(μ_3_-adeninato)_2_(μ-Hadip)_2_]}_n_, carried out at different concentrations of SDS and CTAB, shows that at low concentration, 1 mM, the generated material shows the expected structure corresponding to {[Cu_2_(μ_3_-adeninato)_2_(μ-Hadip)_2_]}_n_ as confirmed by X-ray powder diffraction (Figures S3–S6), while higher concentrations of ionic surfactants produce side reactions, as verified spectroscopically and by X-ray diffraction giving rise to a mixture of products. However, in the case of the use of P_123_, X-ray powder diffraction confirms the structure {[Cu_2_(μ_3_-adeninato)_2_(μ-Hadip)_2_]}_n_, which is not altered independently of the P_123_ concentration (Figure 9 and Figure S7).

Therefore, we focus on the synthesis of the material at low ionic surfactant concentrations to avoid an excess of the surfactant and side reactions. In particular, the reaction carried out with 1 mM SDS produced a decrease in the Z value with no significant change in the particle size to that obtained in experiment c (Table 2, Figure S3). However, the reaction carried out with 1 mM CTAB produces an increase in the pH value, up to 6.9, and an enhance in the Z value which favours the particle aggregation, giving rise to a remarkable increase in the overall particle size to ca. 3 microns but retaining the shape (Table 2, Figure S5).

Finally, the reaction carried out with the neutral P_123_ surfactant, at 1 g/L, does not produce any significant change in the Z potential value, generating octahedral sub-microparticles (Table 2 and Figure S7). Above the critical surfactant concentrations (5 and 15 mg/mL), P_123_ decreases the size of the particles, probably due to it having terminal OH groups (Figure S7) which may favour particle-surfactant hydrogen bond interactions with the free adipic acid carboxylic groups present in compound **1n**, which results in a decrease in the particle-particle interactions (Table 2).

### 3.3. Magnetic Properties 

The thermal variation of the product of the molar magnetic susceptibility versus the temperature per Cu dimer shows very similar results for both samples (**1m** and **1n**) (Figure S8) with a strong antiferromagnetic Cu-Cu interaction. These results are very similar to those found in the crystalline sample [26] and confirm the presence of the same type of Cu(II) dimers with the same structure. We can also observe that there is no a noticeable change in the magnetic parameters in the range studied when the size of the nanoparticles are reduced (Figure S8).

## 4. Conclusions

Bio-inspired CPs present potentially outstanding applications in medical, pharmacological, and cosmetics fields. These applications require the formation of stability to physiological conditions and well-controlled sub-micron and/or nanoparticle sizes. Therefore, studies focusing on size reduction to produce homogeneous particles with defined morphologies are necessary.

In this work, we have selected a copper(II) coordination complex based on adenine, a potentially biocompatible ligand, with molecular recognition capabilities thanks to the presence of free amino and carboxyl groups in its structure.

The control of the reaction parameters, such as reaction temperature, allows the reduction of the particle size from the micron to the sub-micron scale. Interestingly, the reactions carried out in the presence of selected surfactants show remarkable phenomena. Thus, the presence of controlled amounts of neutral surfactant P_123_, with terminal OH groups in its structure, allows the reduction of the particle size to a few hundreds of nanometres, probably due to CP-surfactant H-bond interactions between the OH groups and the free carboxylic groups present in {[Cu_2_(μ_3_-adeninato)_2_(μ-Hadip)_2_]}_n_, while the use of cationic surfactants such as CTAB produces an enhance in the particle size going to the micron scale due to the ionic character of {[Cu_2_(μ_3_-adeninato)_2_(μ-Hadip)_2_]}_n_.

Finally, the studies carried out in water at different pH values show that the obtained sub-micron CP particles are stable over long periods of time and under physiological pH conditions, without changing their size and morphology. These are the conditions required for further use of this CP under any potential biological application.

These studies show the variety of synthetic factors, from a bottom-up approach, which can be used to modify the particle size of CPs and how the CP design can produce materials of potential interest for health applications.

## Figures and Tables

**Table 1:** Reaction conditions to get {[Cu_2_(μ_3_-adeninato)_2_(μ-Hadip)_2_]}_n_ and particle size at pH 5.8.

Experiment	T (°C)	Reaction Time (min)	Adenine (M)	Cu(NO_3_)_2_·3H_2_O (M)	Adipic Acid (M)	Particle Size ^1^ (nm)
*a*	90	15	0.010	0.040	0.040	1200 ± 255
*b*	40	15	0.015	0.040	0.040	381 ± 63
*c*	40	5	0.015	0.040	0.040	403 ± 130
*d*	40	5	0.015	0.050	0.040	470 ± 117
*e*	40	5	0.015	0.050	0.050	340 ± 70
*f*	40	5	0.040	0.040	0.040	521 ± 190
*g*	40	5	0.040	0.080	0.040	580 ± 107
*h*	40	5	0.040	0.040	0.080	465 ± 170

^1^ The means and standard deviations were calculated from values corresponding to 60 particles from SEM images.

**Table 2:** Influence of different surfactants in the dimensions of {[Cu_2_(μ_3_-adeninato)_2_(μ-Hadip)_2_]}_n_.

Surfactant	Z-Potential (mV)	pH	Surfactant Type	Surfactant Concentration	Particle Size ^1^ (nm)
No surfactant	17	5.8	-	-	403 ± 130
SDS	8	5.6	Anionic	1 mM	422 ± 55
CTAB	42	6.9	Cationic	1 mM	2701 ± 265
P123	21	5.8	Neutral	1 g/L	524 ± 212
P123	-	-	Neutral	5 g/L	283 ± 81
P123	-	-	Neutral	15 g/L	296 ± 68

^1^ The means and standard deviations were calculated from values corresponding to 60 particles from the SEM images.

## Supplementary Materials

The following are available online at www.mdpi.com/2073-4360/9/11/565/s1. Table S1: IR selected data of [Cu_2_(*μ*-N3,N9-adeninato)_4_(H_2_O)_2_]·5H_2_O, Figure S1: FESEM images showing the stability of **1n** following **c** experimental conditions, versus pH: (a) pH = 5 and (b) pH = 9, Figure S2: (a) Sodium dodecylsulfate (SDS), (b) cetyltrimethylammonium bromide (CTAB), and (c) a block copolymer of polyethylene glycol (P_123_) used in this reaction. Table S2: Hydrodynamic radius, Z potential and pH values of the different surfactants (SDS, CTAB, and P_123_), in absence of **1n**, Figure S3: FESEM images of sub-microparticles obtained heating at 40 °C, 5 min and leaving the solution 20 h at 25 °C of compound **1n** (a) and the obtained products in the presence of SDS, at 1 mM (b) 5 mM, (c), and 15 mM (d), Figure S4: X-ray powder diffraction patterns of {[Cu_2_(μ_3_-adeninato)_2_(μ-Hadip)_2_]}_n_ (**1n**) (black), **1n** with SDS 1 mM (red), **1n** with SDS 5 mM (blue) and **1n** with SDS 15 mM (green), Figure S5: FESEM images of sub-microparticles obtained heating at 40 °C, 5 min and leaving the solution 20 h at 25 °C of compound **1n** (a) and the obtained products in the presence of CTAB, at 1 mM (b), 5 mM (c), and 15 mM (d), Figure S6: X-ray powder diffraction patterns of {[Cu_2_(μ_3_-adeninato)_2_(μ-Hadip)_2_]}_n_ (**1n**) (black). **1n** with CTAB 1 mM (red). **1n** with CTAB 5 mM (blue) and **1n** with CTAB 15 mM (green), Figure S7: FESEM images of sub-microparticles obtained heating at 40 °C, 5 min and leaving the solution 20 h at 25 °C of compound **1n** (a) and the obtained products in the presence of P_123_, at 1 mM (b), 5 mM (c), and 15 mM (d), Figure S8: Thermal dependence of χ_m_ per Cu(II) dimer for {[Cu_2_(μ_3_-adeninato)_2_(μ-Hadip)_2_]}_n_ as microcrystals (**1m**) and nanocrystals (**1n**). The inset shows the thermal variation of the χ_m_ T product. The solid lines are the best fit to the S = ½ dimer model.
