# Supplementary material for: Direct Formation of Sub-Micron and Nanoparticles of a Bioinspired Coordination Polymer Based on Copper with Adenine
Source: Polymers (Basel). 2017 Nov 1;9(11):565. doi: 10.3390/polym9110565 (PMC6418955; doi:10.3390/polym9110565)
Supplement: Supplementary file 1 [file polymers-09-00565-s001.pdf]

Supporting Information

Direct formation of sub-micron and nano-particles of a bioinspired coordination polymer based on copper with adenine.

Verónica G. Vegas [1], Marta Villar-Alonso[1], Carlos J. Gómez-García[2], Félix Zamora [1,3,4*], Pilar Amo-Ochoa[1,4*]

[1] Departamento de Química Inorgánica, Universidad Autónoma de Madrid, 28049 Madrid, Spain; pilar.amo@uam.es

[2] Departamento de Química Inorgánica. Instituto de Ciencia Molecular (ICMol). Parque Científico. Universidad de Valencia. Catedrático José Beltrán, 2. 46980 Paterna Valencia (Spain). Universidad de Valencia

[3] Instituto de Física de la Materia Condensada (IFIMAC), Universidad Autónoma de Madrid, 28049 Madrid, Spain.

[4] Institute for Advanced Research in Chemical Sciences (IAdChem), Universidad Autónoma de Madrid, 28049 Madrid, Spain.

CONTENT

S1. Structural Characterization

S2. Morphological Studies

S2.1. Morphological stability studies with the time and the pH.

S2.2. Morphological and structural stability studies of 1n with diferent surfactants at different concentrations.

S3. Magnetic Studies

S1. Structural Characterization

Table S1. IR selected data of [Cu_2(μ-N3,N9-adeninato)$_4$(H_2O)$_2$]·5H_2O.

[Cu_2(*μ*-N3,N9-adeninato)$_4$(H_2O)$_2$]·5H_2O Wavenumber (cm^{-1})	Reaction Intermediate Wavenumber (cm^{-1})
3388 (w, br)	3332 (w)
3207 (w, br)	3189 (w)
1636 (s)	1639 (s)
1559 (m)	1540 (s)
1461 (m)	1466 (m)
1399 (m)	1397 (s)
1305 (w)	1308 (m)
1271 (w)	1278 (m)
1192(w)	1209 (s)
1149 (w)	1145 (m)
1030 (m)	1042 (w)
976(w)	992 (w)
940(w)	942 (w)
888 (w)	883 (w)
794 (w)	795 (w)
736 (w)	736 (w)
654 (w)	652 (w)

Morphological Studies

S2.1. Morphological stability studies with the pH

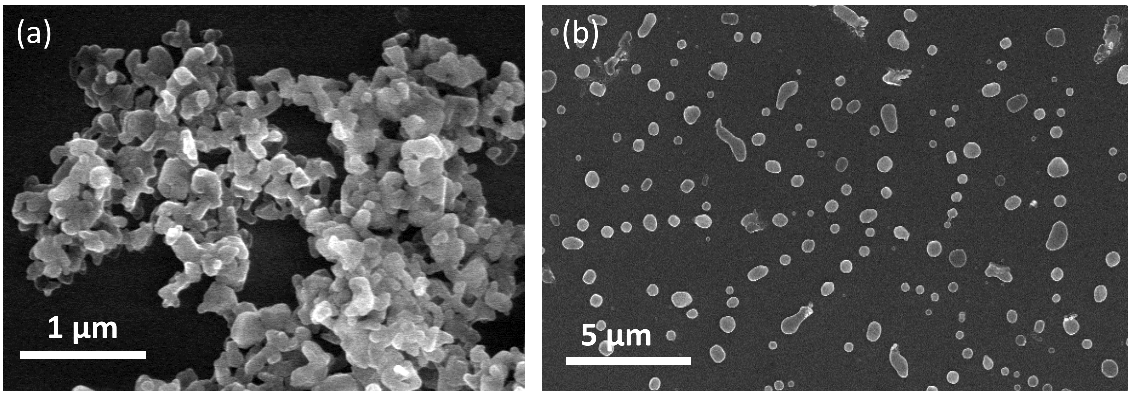

Figure S1. FESEM images showing the stability of **1n** obtained in experiment *c versus* pH: (a) pH=5 and (b) pH=9.

S2.2. Morphological and structural stability studies of 1n, with diferent surfactants at different concentrations.

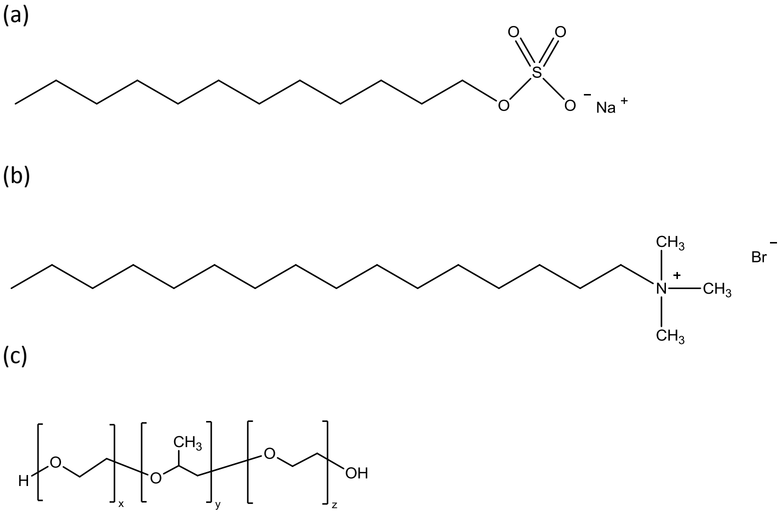

Figure S2. (a) Sodium dodecyl sulfate (SDS), (b) cetyltrimethylammonium bromide (CTAB) and (c) a block copolymer of polyethylene glycol (P123) used in this reaction.

Table S2. Hydrodynamic radius, Z potential and pH values of the different surfactants (SDS, CTAB and P_{123}), in absence of **1n.**

Surfactant	pH	Hydrodynamic Radius (nm)	*Z* Potential (mV)
SDS	7.87	288	-75.1
CTAB	8.75	146.9	71.9
P_{123}	8.24	22.2	-4.7

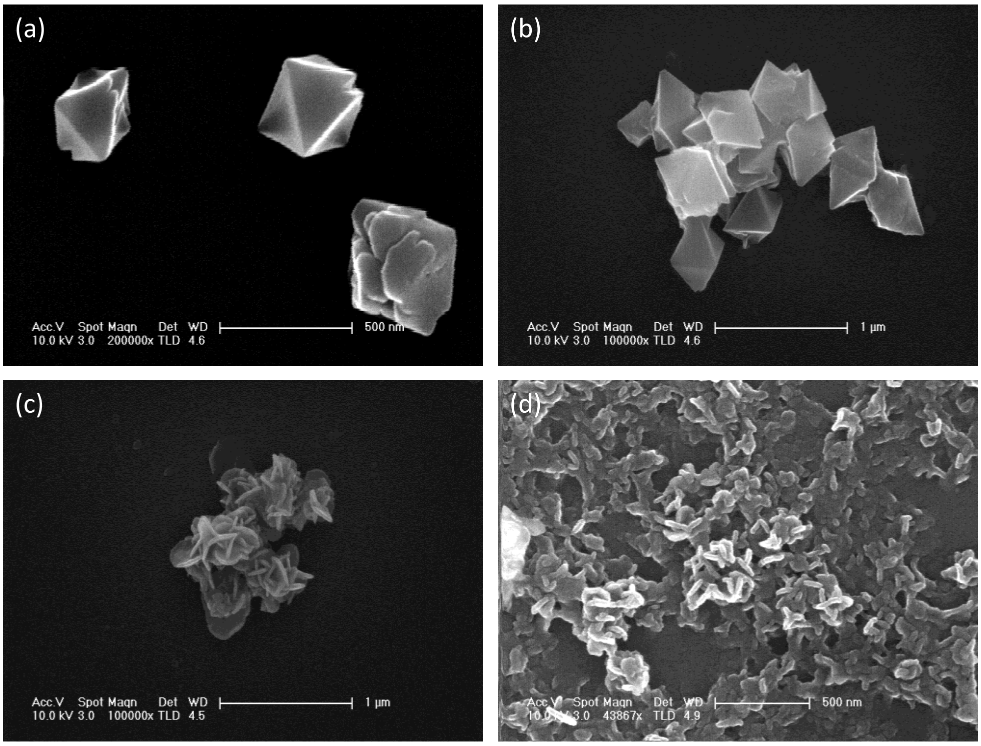

Figure S3. FESEM images of sub-microparticles obtained heating at 40 °C, 5 minutes and leaving the solution 20 h at 25 °C of compound **1n** (a) and the obtained products in the presence of SDS, at 1 mM (b) 5 mM, (c) and 15 mM. (d).

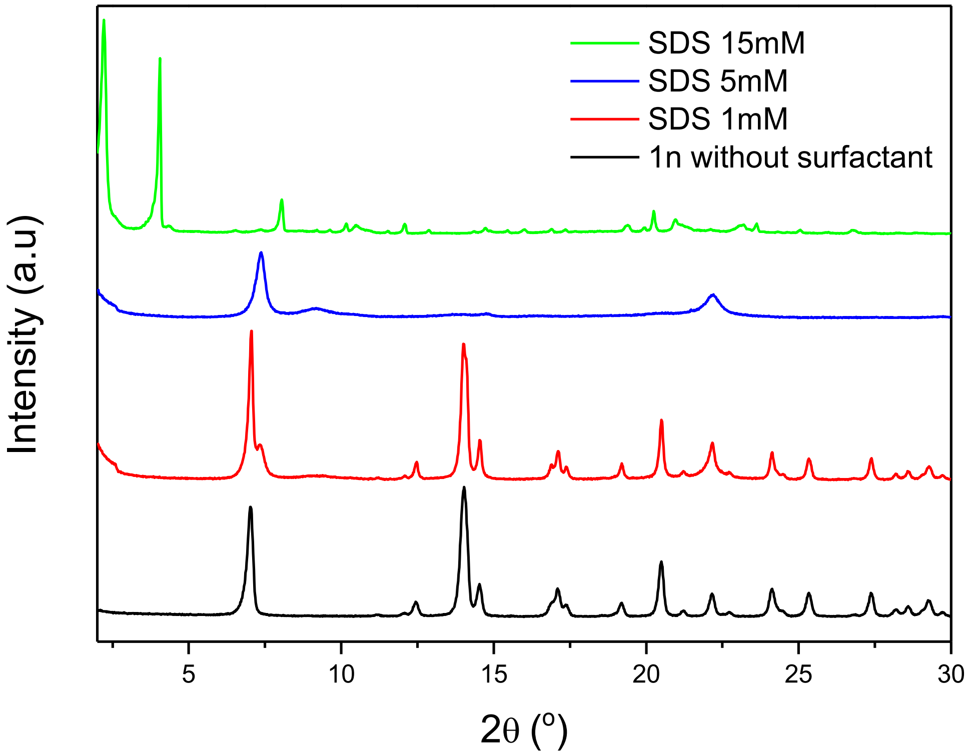

Figure S4. X-ray powder diffraction patterns of {[Cu_2(μ_3-adeninato)$_2$(μ-Hadip)$_2$]} (**1n**) (black line). Red line corresponds to **1n** with SDS 1mM, blue line to **1n** with CTAB 5 mM and green line to **1n** with SDS 15 mM.

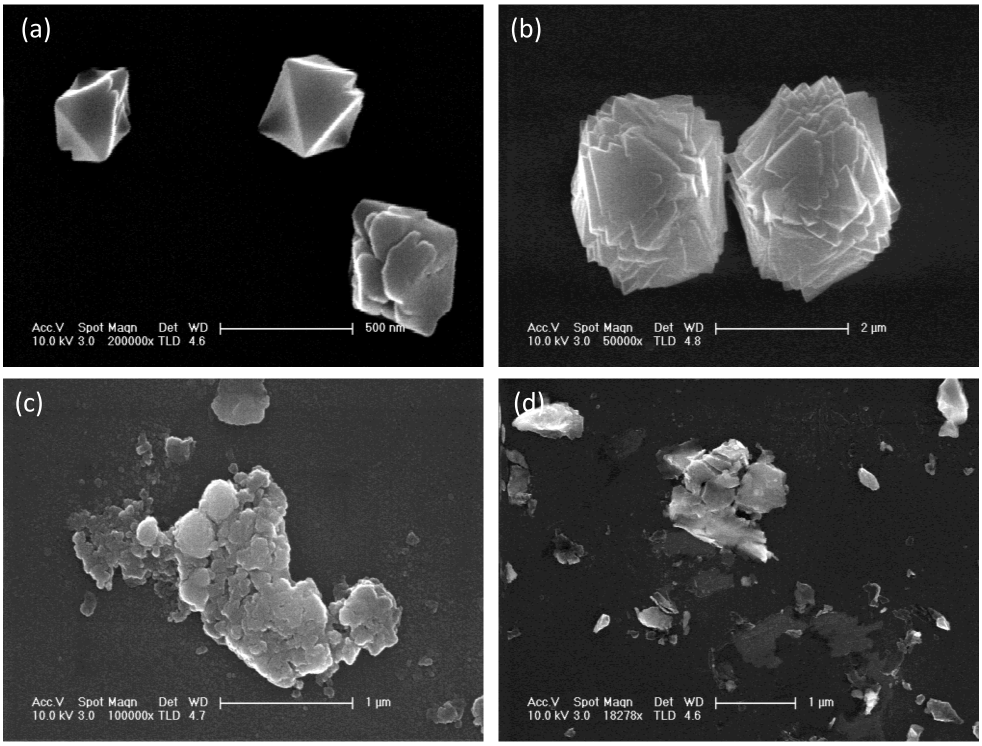

Figure S5. FESEM images of sub-microparticles of compound **1n** obtained heating at 40 °C, 5 min. and allowing to stand the solution for 20 h at 25 °C (a) and the obtained products in the presence of CTAB, at 1 mM (b), 5 mM, (c) and 15mM (d).

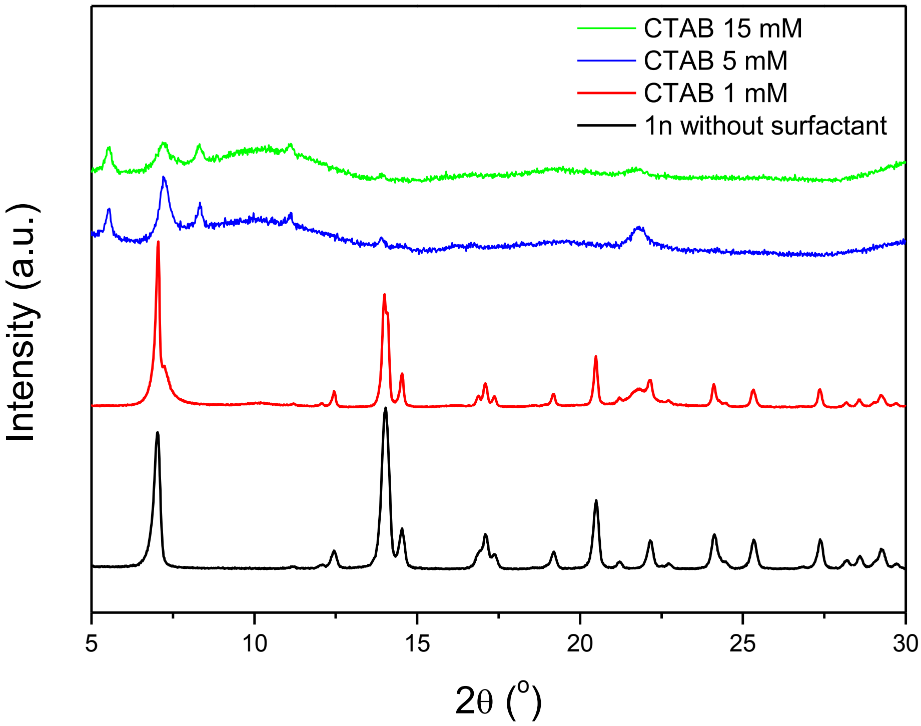

Figure S6. X-ray powder diffraction patterns of {[Cu_2(μ_3-adeninato)$_2$(μ-Hadip)$_2$]}$_n$ (**1n**) (black line). Red line corresponds to **1n** with CTAB 1 mM, blue line to **1n** with CTAB 5 mM and green line to **1n** with CTAB 15 mM.

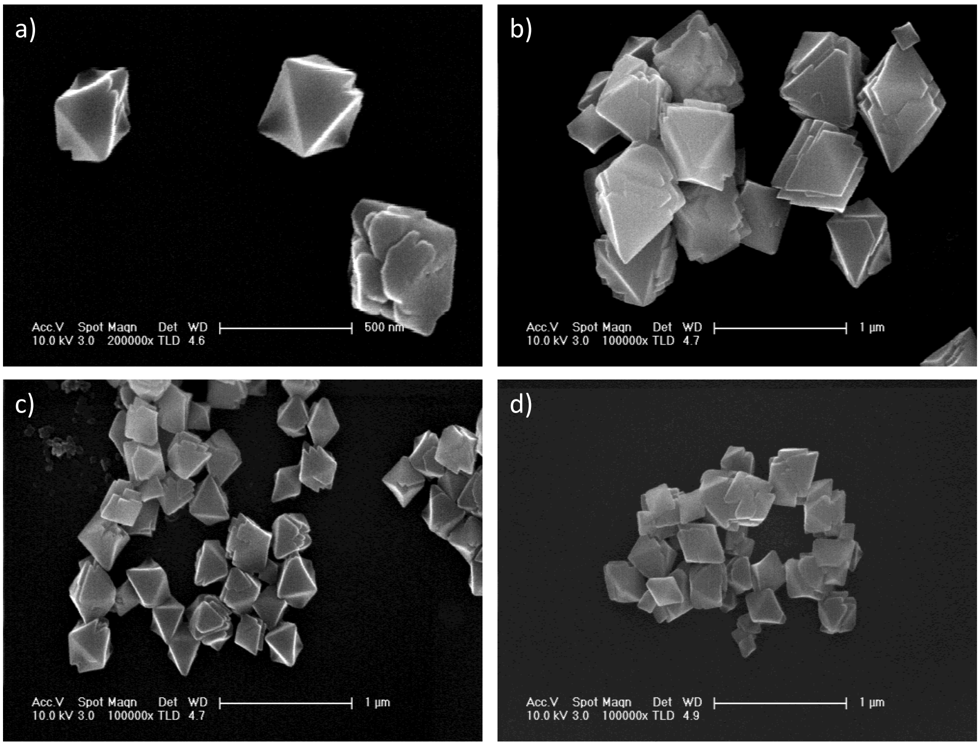

Figure S7. FESEM images of sub-microparticles of compound **1n** obtained heating at 40 °C, 5 min. and allowing to stand the solution for 20 h at 25 °C (**a**) and the obtained products in the presence of P_{123}, at 1 g/L (**b**) 5 g/L (**c**) and 10 g/L (**d**).

S3. Magnetic Studies

At room temperature the $\chi_m T$ value is *ca*. 0.5 cm^3 K mol^{-1}, a value well below the expected one for two non-interacting Cu(II) ions (0.375 x 2 = 0.75 cm^3 K mol^{-1} for g = 2). This low value suggests the presence of a strong antiferromagnetic Cu-Cu interaction. When the two samples are cooled, the $\chi_m T$ value decreases a reaches a value very close to zero below *ca*. 50 K (inset in Figure S8). This behaviour confirm the presence of a strong intradimer antiferromagnetic coupling between the Cu(II) ions. A further confirmation of this behaviour is provided by the presence of a rounded maximum in the thermal variation of the molar magnetic susceptibility (Figure S8). The presence of a small divergence in c_m at very low temperatures indicates the presence of a very small paramagnetic monomeric impurity that may be attributed to crystal defect and Cu(II) vacancies in a small fraction of dimers. Since the structure of compounds **1m** and **1n** show the presence of Cu(II) dimers connected through a double carboxylato and double adeninato bridges, we have fit the magnetic properties to a simple S = ½ dimer model. We consider in a first approximation, that the N7-adenine-N9 bridge can be neglected when compared with the cuadruple bridge connecting the two Cu(II) ions in the dimer. This approximation is justified by previous results with this kind of adeninato bridge where weak AF couplings have been found.[1-4]. We have included a paramagentic monomeric impurity in the model (c) to reproduce the divergence in c_m at very low temperatures. This simple model reproduces very satisfactorily the magnetic properties of **1m** and **1n** with the following

parameters: g = 2.066, J = -241 cm^{-1} and c = 1.2 % for **1m** and g = 2.088, J = -254 cm^{-1} and c = 1.3 % for **1n** (solid lines in Figure S8).

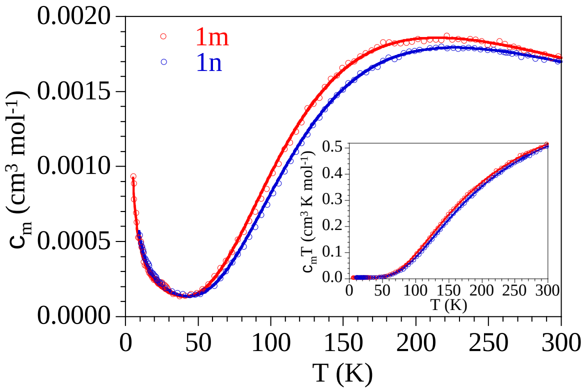

Figure S8. Thermal dependence of χ_m per Cu(II) dimer for *{[Cu$_2$(μ_3-adeninato)$_2$(μ-Hadip)$_2$]}$_n$* as microcrystals (**1m**) and nanocrystals (**1n**). Inset shows the thermal variation of the χ_mT product. Solid lines are the best fit to the S = ½ dimer model.

References

1. Asakawa, T.; Inoue, M.; Hara, K.; Kubo, M. Magnetic Properties of Copper(II) Complexes of 6-Aminopurine and 6-Hydroxypurine. *Bull. Chem. Soc. Jpn.* **1972**, *45*, 1054-1057.
2. Pérez-Yáñez, S.; Beobide, G.; Castillo, O.; Cepeda, J.; Luque, A.; Román, P. Directing the Formation of Adenine Coordination Polymers from Tunable Copper(II)/Dicarboxylato/Adenine Paddle-Wheel Building Units. *Cryst. Growth Des.* **2012**, *12*, 3324-3334.
3. García-Terán, J.P.; Castillo, O.; Luque, A.; García-Couceiro, U., Román, P.; Lezama, L. An Unusual 3D Coordination Polymer Based on Bridging Interactions of the Nucleobase Adenine. *Inorg. Chem.* **2004**, *43*, 4549-4551.
4. Pérez-Yáñez, S.; Beobide, G.; Castillo, O.; Cepeda, J.; Luque, A.; Aguayo, A.T.; Román, P. Open-Framework Copper Adeninate Compounds with Three-Dimensional Microchannels Tailored by Aliphatic Monocarboxylic Acids. *Inorg. Chem.* **2011**, *50*, 5330-5332.
